# Proteomics Profiling of KAIMRC1 in Comparison to MDA-MB231 and MCF-7

**DOI:** 10.3390/ijms21124328

**Published:** 2020-06-18

**Authors:** Bandar Alghanem, Rizwan Ali, Atef Nehdi, Hajar Al Zahrani, Abdulelah Altolayyan, Hayat Shaibah, Omar Baz, Alshaimaa Alhallaj, James J. Moresco, Jolene K. Diedrich, John R. Yates, Mohamed Boudjelal

**Affiliations:** 1 Medical Research Core Facility and Platforms (MRCFP), King Abdullah International Medical Research Center/King Saud bin Abdulaziz University for Health Sciences (KSAU-HS), King Abdulaziz Medical City (KAMC), NGHA, Riyadh 11426, Saudi Arabia; GhanemBa@NGHA.MED.SA (B.A.); aliri@ngha.med.sa (R.A.); nehdiat@NGHA.MED.SA (A.N.); alzahraniha6@NGHA.MED.SA (H.A.Z.); altolayyanab@NGHA.MED.SA (A.A.); ShaibahHa@NGHA.MED.SA (H.S.); bazom@NGHA.MED.SA (O.B.); alhallajal@NGHA.MED.SA (A.A.); 2 Department of Molecular Medicine, The Scripps Research Institute, La Jolla, CA 92037, USA; jmoresco@scripps.edu (J.J.M.); jdiedric@scripps.edu (J.K.D.); jyates@scripps.edu (J.R.Y.III)

**Keywords:** breast cancer, cell lines, TMT, mass spectrometry, proteomics, phosphoproteomic

## Abstract

Proteomics characterization of KAIMRC1 cell line, a naturally immortalized breast cancer cells, is described in comparison to MCF-7 and MDA-MB-231 breast cancer cells. Quantitative proteomics analysis using the tandem mass tag (TMT)-labeled technique in conjunction with the phosphopeptide enrichment method was used to perform comparative profiling of proteins and phosphoproteins in the three cell lines. In total, 673 proteins and 33 Phosphoproteins were differentially expressed among these cell lines. These proteins are involved in several key cellular pathways that include DNA replication and repair, splicing machinery, amino acid metabolism, cellular energy, and estrogen signaling pathway. Many of the differentially expressed proteins are associated with different types of tumors including breast cancer. For validation, 4 highly significant expressed proteins including S-methyl-5′-thioadenosine phosphorylase (MTAP), BTB/POZ domain-containing protein (KCTD12), Poly (ADP-ribose) polymerase 1 (PARP 1), and Prelamin-A/C were subjected to western blotting, and the results were consistent with proteomics analysis. Unlike MCF-7 and MDA-MB-231, KAIMRC1 showed different phospho- and non-phosphoproteomic phenotypes which make it a potential model to study breast cancer.

## 1. Introduction 

Breast cancer is known to be the most common cause of death in women in the west after lung cancer [1]. Records from Europe revealed that the main cause of cancer morbidity and mortality in women is breast cancer [2]. In Saudi Arabia, there is a significant increase in breast cancer incidences from 2008 to 2010 with an alarming 21% growth rate [3]. Even though major in-roads have been made into both the understanding and treatment of this disease, we still lack a complete comprehension of breast cancer etiology. Furthermore, several models such as animals, biopsies, and cell lines are being used to study breast cancer biology and to predict human response to different types of therapy. Each one of these models has its own pros and cons; however, due to the cost-effectiveness, simplicity of handling, and unlimited self-replicating source, cell lines are preferred over other models [4]. The majority of breast cancer studies performed so far have used well-established disease-relevant cell lines such asMCF-7 and MDA-MB-231 [5,6]. These cell lines are used for decades due to their perceived stability in culture; however, whether they truly represent what happens in a breast tumor is debatable. Moreover, most of these cell lines originated from Caucasian patients. To this end, several novel breast cancer cell lines have been established from primary tumors and different ethnic groups in order to be more representative of breast cancer models [7,8]. Choosing the right cell line to study breast cancer is crucial to represent the heterogeneity of this disease. Moreover, establishing new breast cancer cell lines from different ethnic groups might shed more light on specific pathways involved in breast cancer and it can also contribute to improving the current treatment procedures. 

Characterization of these cell models using either genomics or transcriptomics-based approaches provides important information regarding the biology underpinning their tumorigenicity. However, these approaches are limited as they do not provide much information at the cellular function level. This requirement can be fulfilled using a quantitative proteomics-based approach that can provide quantitative and qualitative (phosphorylation) measurements of proteins at any given cell state. Furthermore, Geiger et al. showed that there is a poor correlation between protein expression and copy number of genes in cancer cells, which indicates that gene expression variations do not necessarily translate to protein expression [9]. On the other hand, Sacco et al. showed a lack of correlation between transcriptomics and proteomics in response to the drug in the MCF-7 cell line [10]. Taking all this together, proteomics analysis is an essential and complementary approach to genomics/transcriptomics and has a fundamental impact on studying cell lines. 

Our group previously isolated and characterized a novel naturally immortalized breast cancer cell line, KAIMRC1, from a Saudi Arabian female breast cancer patient after surgical biopsy [11]. Interestingly, in these cells, the gene expression profile of several key genes implicated in breast cancer DNA repair, signal transduction, and cell cycle were found to be altered. These exciting results motivated us to further look into the proteome profile of KAIMRC1 cells that might open new avenues towards breast cancer biomarkers and drug discovery. Thus, in this study, we applied a comprehensive proteomics analysis in conjunction with appropriate bioinformatics tools to study this cell line. Here, we performed a Tandem Mass Tag (TMT) strategy for accurate peptide/protein quantification [12]. Proteomics profiling was carried out on KAIMRC1 cells versus other breast cancer cell lines such as MCF-7 and MDA-MB-231. Furthermore, the phosphoproteomics profiles were compared as well. 

## 2. Results and Discussion 

**Figure 1:**
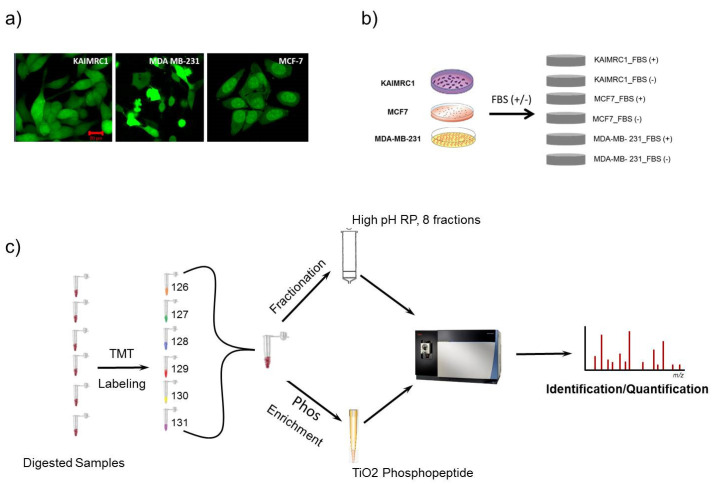
Schematic description of the experimental design employed in proteomics analysis: (**a**) KAIMRC1, MCF-7, and MDA-MB-231 cells stained for cytoplasm (Green) with Cytotracker^®^ Green. (**b**) Three cell lines, KAIMRC1, MCF-7, and MDA-MB-231, were stimulated with or without Fetal Bovine Serum (FBS); (**c**) quantitative proteomics analysis workflow using a tandem mass tag (TMT) strategy with High pH Reversed-Phase Peptide Fractionation technique and TiO_2_ phosphopeptide enrichment methods.

To identify differentially expressed proteins using Mass Spectrometry (MS), the initial step of the study was to carry out proteome and phosphoproteome profiling on newly established breast cancer cells “KAIMRC1”. This profiling was performed in comparison with other commonly used cell lines in breast cancer research: MCF-7, an estrogen/progesterone-positive, human epidermal growth factor receptor 2 (HER2)-negative cell line, and MDA MB-231, an aggressive triple-negative cell line (Figure 1a). These two cell lines represent different molecular subtypes and stages of breast cancer, and that was the main reason for selecting them to benchmark with KAIMRC1. Moreover, the characterization of KAIMRC1 cells showed distinct morphology, cell surface markers, and growth characteristics comparable to both MCF-7 and MDA-MB-231 cell lines [11]. 

In normal culturing condition (complete media), different cells, especially when they have the same tissue origin, do not demonstrate significant detectable differences in term of protein expression and phosphorylation, but when submitted under a physiological stress (serum starvation, hypoxia, oxidative stress, etc.), their behavior (gene expression) will be tightly dependent on their specific genetic and metabolic alterations. For a better characterization of their protein profiles and their metabolic reaction to physiological stress, total proteins were extracted from KAIMRC1, MDA-MB-231, and MCF-7 cells grown in normal conditions or under serum starvation for 24 h (Figure 1b). For accurate proteomics quantitative analysis and in order to increase the detection of proteins in samples, we utilized TMT-labeled strategy in combination with high pH reversed-phase peptide fractionation chromatography [13,14]. For phosphoproteomics analysis, TiO_2_ phosphopeptide enrichment spin tips were used after TMT labeling to increase the signal-to-noise ratio of endogenous phosphopeptide in digested samples [15]. Illustration of the workflow that was followed in this study is shown in Figure 1c. These analyses generated a total of 6068 identified proteins and 748 phosphoproteins with false discovery rate (FDR) ≤ 1%. The phospho-site localization was determined based on Ascore approach [16] as shown in Supplementary Tables S1 and S2. The number of identified proteins was varying among all the cell lines with a total of 5803, 5993, and 5747 proteins and 617, 674, and 545 phosphoproteins for KAIMRC1, MCF-7, and MDA-MB-231, respectively (Table 1). The identified proteins and phosphoproteins in all three cell lines were grouped based on their class using the PANTHER system [17] as presented in supplementary Figure S1. The majority of proteins/phosphoproteins in all the three cell lines were found to be in the category of nucleic acid-binding proteins, hydrolases, and enzyme modulators.

**Figure 2:**
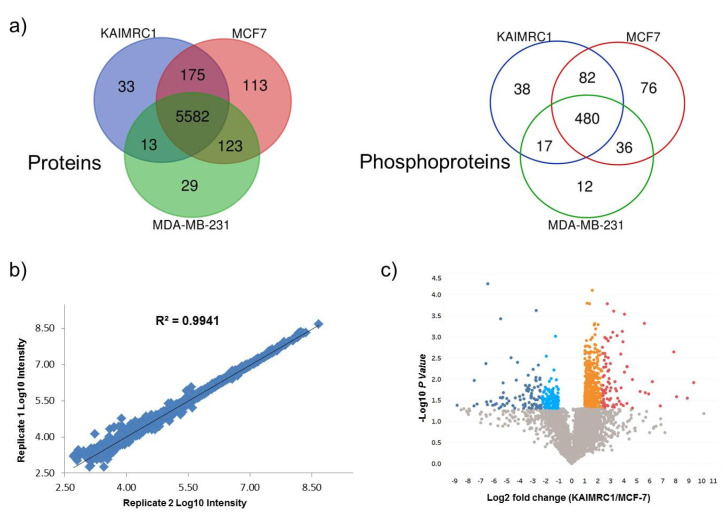
Quantitative proteomics analysis: (**a**) Venn diagram illustrated the overlap identified protein (left) and phosphoproteins (right) between KAIMRC1, MCF-7, and MDA-MB-231. (**b**) Reproducibility evaluation for duplicate biological replicates in MCF7 cell lines using proteins normalized intensities (Reproducibility evaluation of proteins and phosphoproteins for other cell lines can be found in supplementary Figures S2 and S3). (**c**) Volcano plot display differentially expressed proteins in KAIMRC Versus MCF7: Colored circles are significant proteins (*p* < 0.05), and red is highly upregulated proteins with fold change ≥ 5. Upregulated protein is plotted in orange with fold change between 2 to 5. Light blue is downregulated protein with fold change ≤ 0.5. Dark blue is highly downregulated protein with fold change ≤ 0.2. Gray circles are nonsignificant proteins. (Volcano plots of differentially expressed proteins and phosphoproteins for the other pairwise comparisons showed in Supplementary Figures S4 and S5).

Comparative results revealed that 92% of the identified proteins are common among the three cell lines while only ~65% of the phosphoproteins overlap (Figure 2a). The eminent number of common proteins/phosphoproteins reflects the similarity of the proteome of the three breast cancer cell lines. However, our prime interest was to investigate the differentially expressed proteins (DEPs) in newly isolated cell line KAIMRC1. This comparative study of KAIMRC1 was performed to shed more light on dysregulated pathways involved in the transformation and the spontaneous immortalization of these cells. Thus, we applied pairwise comparisons for KAIMRC1 versus MCF-7 and MDA-MB-231. First, we evaluated the reproducibility of the proteomic quantitative analysis based on normalized reporter intensities using duplicate biological samples of KAIMRC1, MCF-7, and MDA-MB-231 cultured separately on different days. Results showed excellent reproducibility with an average of r^2^ > 0.96 for proteins and r^2^ > 0.84 for phosphoproteins as illustrated in Figure 2b. Reproducibility evaluation of proteins and phosphoproteins for other cell lines can be found in supplementary Figures S2 and S3. A previously published study using TMT-10-plex experiments has shown a similar degree of reproducibility [18]. 

The DEP measurements were set with fixed statistical criteria with estimated fold change ≥ 2 for upregulation and ≤0.5 for downregulation with *p* < 0.05. The quantitative results have been demonstrated by constructing a volcano plot as shown in the pairwise comparison of KAIMRC versus MCF-7 (Figure 2c). Volcano plots of differentially expressed proteins and phosphoproteins for the other pairwise comparisons are shown in Supplementary Figures S4 and S5. Pairwise comparison of KAIMRC1/MCF-7 cultured under normal conditions resulted in 760 DEPs. Among these DEPs, 321 (42.3%) proteins were found to be downregulated, while 439 (57.7%) were upregulated. In contrast, in serum-starved cells, DEPs were reduced to 707 with 208 (29.4%) downregulated proteins and 499 (70.6%) upregulated proteins. Results showed that 235 of DEPs were shared between cells in normal and starvation condition. The comparison of phosphoproteins for KAIMRC1/MCF-7 was similarly investigated. A total of 37 phosphoproteins were differentially expressed; 26 were found to be downregulated in KAIMRC1 and 11 were upregulated. In serum-starved cells, 47 phosphoproteins showed differential expression; 16 of them were downregulated in KAIMRC1 and the rest were upregulated. A similar pairwise analysis was conducted to compare KAIMRC1 to MDA-MB-231. Under normal culturing conditions, 522 DEPs were identified in which 173 were downregulated and 349 were upregulated. In term of phosphoproteins, only 11 were differentially expressed in that 6 phosphoproteins were downregulated and 5 were upregulated. Interestingly, serum-starved KAIMRC1 and MDA-MB-231 showed an increase in the number of DEPs, with a total of 703 proteins; 269 were found to be downregulated, while 434 were upregulated. Nevertheless, for phosphoproteins, there has been a remarkable increase that goes beyond 72% with a total number of 39 differentially expressed phosphoproteins, among them 17 were downregulated whereas 22 were upregulated when compared to normal culturing conditions. Supplementary Tables S3 and S4 show all differentially expressed proteins and phosphoproteins for both pairwise comparisons including “KAIMRC1 vs. MCF-7” and “KAIMRC1 vs. MDA-MB-231”. Moreover, the evaluation of proteins and phosphoproteins that were common in the normal and starved conditions showed no culture condition-dependent change in the level of protein expression (data not shown). This result indicates that serum starvation has no effect on the level of expression of the common and significantly expressed proteins. 

We have examined the differentially expressed proteins and phosphoproteins and their involvement in pathway enrichment analysis using DAVID software [19]. Kyoto Encyclopedia of Genes and Genomes (KEGG) database was selected with statistical criteria set at *p* < 0.05 and the pathway terms were ranked based on the fold enrichment. KEGG pathway analysis showed that the differentially expressed proteins in KAIMRC1/MCF-7 were significantly enriched in several pathways during the starvation condition. Results revealed that the upregulated proteins in KAIMRC1 were associated with several key pathways such as DNA replication, mismatch repair, arginine and proline metabolism, lysine degradation, and pyruvate metabolism. These observations could explain the relatively high proliferation rate and starvation endurance of KAIMRC1 cells in comparison to MCF-7 (data not shown). On the other hand, the downregulated proteins were found to be involved interestingly in ECM–receptor interaction and apoptosis-related pathways. ECM is integral in the maintenance of cellular structure and function. The interactions between cells and the ECM are mediated by transmembrane molecules that include integrins and other cell surface-associated proteins. These interactions are actually the basis for cellular adhesion, movement, proliferation, and even apoptosis [20]. KAIMRC1 cells are observed to attach loosely to the cell culture flasks and plates that gel well with our latest finding that proteins involved in the ECM–receptor interaction pathway are downregulated. For example, integrin beta-1 and syndecan-4 have been found to be highly downregulated in KAIMRC1. There is also a strong possibility that the downregulation of proteins involved in apoptotic regulation might be linked to the spontaneous immortalization and transformation of KAIMRC1. Experiments are underway to substantiate our findings.

Pathway enrichment analysis was performed as well for differentially expressed proteins in KAIMRC1/MDA-MB-231. Results revealed that, during serum-starvation conditions, the upregulated proteins in KAIMRC1 are involved in metabolic pathways including DNA replication; cysteine and methionine metabolism; splicing machinery; valine, leucine, and isoleucine degradation; and mRNA surveillance. All of the abovementioned pathways are directly involved in cell growth and survival. This data confirmed the relatively aggressive phenotype (proliferation and migration invasion) of KAIMRC1 described in our previous study [11]. 

**Figure 3:**
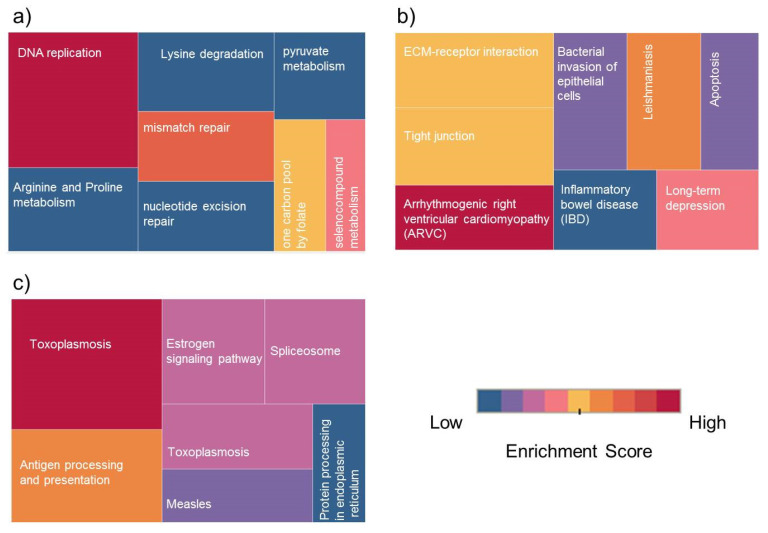
Treemap of Kyoto Encyclopedia of Genes and Genomes (KEGG) pathway classes for differentially expressed proteins (DEPs): Top Enrichment pathway classes for (**a**) upregulated proteins in KAIMRC1 versus MCF-7, (**b**) downregulated proteins in KAIMRC1 versus MCF-7, and (**c**) upregulated phosphoproteins in KAIMRC1 versus MCF-7 and MDA-MB-231. The color and size of each box indicate enrichment score and protein counts for each group. The enrichment analysis was set with statistical criteria (*p* < 0.05).

Under standard culture conditions, pathways involved in DNA replication and repair and in splicing were also upregulated, which indicates that these pathways are constitutively upregulated in KAIMRC1. In contrast, the ECM–receptor interaction pathway has been found to be downregulated. Figure 3a,b shows an example of pathway categories associated with up- and downregulated proteins in KAIMRC1 compared to MCF-7 during starvation conditions. However, the rest of the pathway terms for other conditions are displayed in supplementary Table S5. Many of the identified pathways are common breast cancer-associated molecular pathways such as DNA replication and mismatch repair. Dysregulation of these pathways usually results in mutations due to DNA damage and replication error, leading eventually to tumor growth [21,22,23]. 

**Figure 4:**
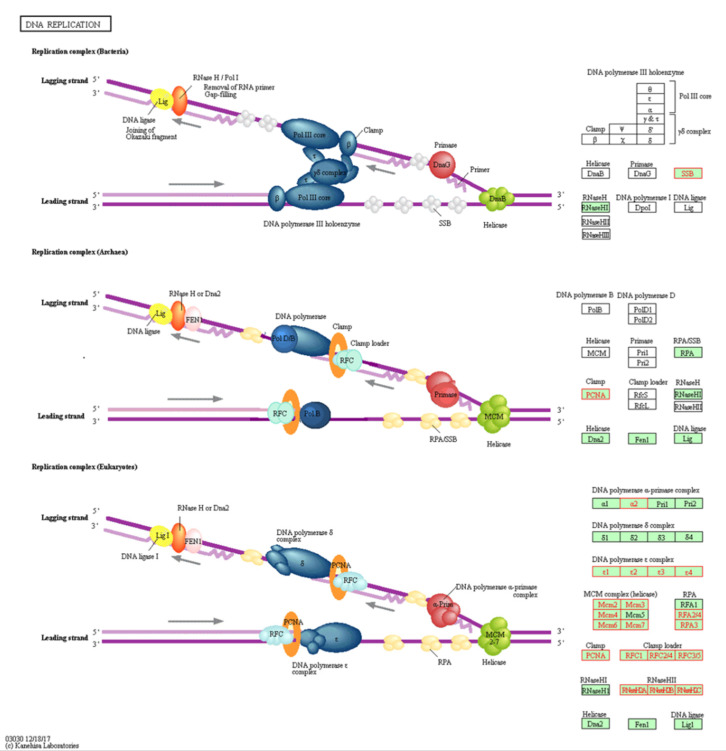
Illustration of DNA replication pathway in KEGG: Genes with red color are associated with upregulated proteins in KAIMRC1 compared to MCF-7 and MDA-MB-231 cell lines. Genes with green or white were not detected.

DNA replication pathway has been found to be the top identified pathway upregulated in the KAIMRC1 cell line. Figure 4 shows all the upregulated proteins in KAIMRC1 that are involved in the DNA replication pathway using KEGG. It was observed that all the DNA polymerase epsilon subunits, RNaseHII, and the majority of minichromosome maintenance protein complex (MCM) was identified in this pathway. All these enzymes are crucial in DNA replication. Altered expression of these enzymes is associated with different types of tumors including breast cancer [24,25,26].

Moreover, some of the identified metabolic pathways including arginine and proline metabolism and lysine degradation have a known role in the energy metabolism of cancer cells [27,28]. In the pairwise comparison of phosphoproteins, a significant difference was observed when cells were serum-starved. This finding was expected and showed that the three analyzed cell lines behave differently to physiological stress. These pathways include antigen processing and presentation, estrogen signaling pathway, and splicing machinery (Figure 3c). It has been previously shown that the expression of antigen processing and presenting molecules are implicated in brain metastasis of breast cancer [29]. Moreover, it is well known that estrogen signaling pathway has an important role in breast cancer metastasis [30]. 

In order to identify potential biomarkers from the differentially expressed proteins list that could be used to discriminate between the three cell lines and possibly different subtypes of breast cancer, we have applied restricted statistical criteria to narrow down our list with a threshold *p* < 0.001 and fold change ≥ 5 for upregulation and ≤0.2 for downregulation. For phosphoproteins, the regulation threshold was set similar but with a *p* < 0.01. The results revealed 33 proteins and 5 phosphoproteins based on these criteria as shown in supplementary Tables S6 and S7 respectively. Among these proteins, one has shown high upregulation in KAIMRC1 cells compared to other cell lines: S-methyl-5′-thioadenosine phosphorylase (MTAP). This protein is an enzyme that has an essential role in polyamine metabolism and is involved in the salvage of adenine and methionine. The deficiency of MTAP results in the accumulation of methylthioadenosine (MTA) that eventually deactivates the adenine and methionine salvage pathway. It has been shown that MTAP-deficiency linked to several soft and solid tumors including breast cancer [31]. Interestingly, it was shown in a previous study the MTAP protein is not detected in MCF-7 and MDA-MB-231 using western blot analysis. This supports our finding where this protein was highly downregulated in these two cell lines using our quantitative proteomics approach [32]. However, the high elevation of this protein in KAIMRC1 shows the differences in the phenotype of these three cell lines.

BTB/POZ domain-containing protein (KCTD12) is another protein that showed high overexpression in KAIMRC1 relative to MCF-7 and MDA-MB-231. This protein is one of the potassium channel tetramerization domain (KCTD) protein families that has a role as an auxiliary subunit of GABAB receptors to alter the emotionality and neuronal excitability [33]. The expression of KCTD12 transcript and protein was associated with multiple tumor types including gastrointestinal stromal, melanoma, and esophageal squamous cell [34,35,36]. Here, we report significant upregulation of KCTD12 in the KAIMRC1 cells in spite of the fact that previous proteomics-based results suggest downregulation of this protein in a breast cancer tissue isolated from a patient with invasive carcinoma [37]. This result gives us another indication of the well-known heterogeneity of breast cancer cells. It also strengthens the idea that tumor formation is not just merely a result of any single mutation or overexpression of single protein but that it is actually a cascade of genes and proteins changing their expression levels resulting in the formation of a tumor. 

Poly (ADP-ribose) polymerase 1 (PARP1) is another protein that showed high significance and overexpression in KAIMRC1 relative to MCF-7 and MDA-MB-231. This protein is a chromatin-associated enzyme that has an important role in DNA damage and repair [38]. The overexpression of PARP1 is implicated in many tumors including colorectal, liver, and ovarian [38,39,40]. However, the inhibition of PARP1 results in repressing tumor growth and metastasis, which make PARP1 inhibitors potential anticancer drugs target that has been suggested by several research studies [41]. There are already several PARP1 inhibitors in the market with promising applications in breast cancer [42].The overexpression of PARP1 in KAIMRC1 cells makes them a potential cell model to study PARP1 activity for the discovery of antitumor drugs. Interestingly, previous studies showed that the PARP1 mRNA and protein expression is elevated in triple-negative breast cancer cell lines. The latter findings also showed that the expression in MDA-MB-231 is higher than in MCF-7, which is consistent with our results. 

Prelamin-A/C is one of the phosphoproteins that showed high significance and downexpression in KAIMRC1 compared to MDA-MB-231. This protein is one of the Lamin family that interacts with chromatin and plays a role in forming nuclear lamina inside the nucleus. Thus, this protein is involved in several biological functions including cell cycle regulation, DNA repair, and DNA replication. In most breast cancer cell lines, a significant fraction of the Lamin A/C-negative population has been observed, which leads to deformed nuclear morphology in cancer cells and aneuploidy. A former study reported that the expression level of Lamin A/C was low in MCF-7 compared to MDA-MB-231 [43]. Our result showed that this protein is phosphorylated on one tyrosine residue (Tyr (19)) and two serine residues (Ser (22) and Ser (392)), which is consistent with previous phosphoproteomics analysis that was performed in human cancer cell [44]. 

**Figure 5:**
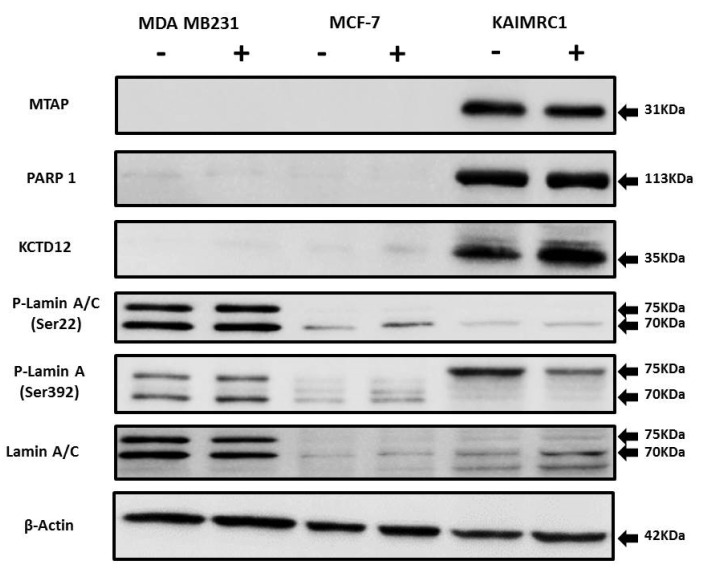
Expression of MTAP, PARP1, KCTD12, and Lamin A/C in cancer cell lines: Western blot analysis of protein expression of MTAP, PARP1, KCTD12, Lamin A/C, and phosphorylated Lamin A/C in MDA MB231, MCF-7, and KAIMRC1 cells. Preparation of cell lysates and western blot were performed as described under the Materials and Methods section. In both normal (+) and serum-starved (-) conditions, KAIMRC1 cells showed strong expression of MTAP, PARP1, and KCTD12 compared to MDA-MB231 and MCF-7 cells. Moreover, KAIMRC1 cells showed weak expression of Lamin A/C and phosphorylated Lamin A/C in comparison to MDA MB231 cells, thus validating our proteomics results.

To further validate our proteomics analysis results, all the identified proteins (MTAP, KCTD12, PARP1, and prelamin-A/C) were subjected to western blotting analysis (Figure 5). There is a notable increase in phosphorylation of Lamin A at Ser 392 in serum starvation condition in the KAIRMC1 cells. Phosphorylation of Lamins is cell-cycle dependent and involved in many cellular processes [45]. Earlier it has been reported that Lamin A accumulation in the nuclear membrane increases during quiescence. Interestingly, Lamin A is also a nuclear target of AKT phosphorylation [46]. It might be possible that Lamin A is highly phosphorylated due to the autophosphorylation of AKT in starvation condition that consequently contributes to resist autophagy during quiescence. It is well documented that serum starvation induces autophagy, an adaptive cell survival mechanism [47]. To further investigate, we looked into our data for the autophagy-related proteins but we did not find any change in the level of expression of these proteins during the starvation condition. This result implies that at least 24-h serum-starvation does not induce autophagy-related cell death in KAIMRC1 cells possibly through AKT-induced suppression of autophagy [48], whereas in the MCF-7 cell line some of the autophagy-related proteins are upregulated, indicating that the process of autophagy is distinctly different among these cell lines.

Nevertheless, western blot results showed similar expression levels as obtained by the proteomics analysis, thus verifying the reliability of the differentially expressed proteins data.

## 3. Materials and Methods 

### 3.1. Materials 

All the chemicals including triethylammonium bicarbonate buffer, 2-chloroacetamide, ammonium acetate, Radioimmunoprecipitation assay (RIPA) buffer, and protease inhibitors were purchased from Sigma-Aldrich (St. Louis, MO, USA). HPLC-grade water, acetonitrile, methanol, and formic acid were acquired from Fisher Scientific (Waltham, MA, USA). Trypsin was purchased from Promega (Madison, WI, USA). bicinchoninic acid (BCA) protein assay, High select TiO_2_ Phosphopeptide Enrichment Kit (Lot number: SF243171), and TMTsixplex™ Isobaric Label Reagent kits (Lot number: SH254566) were purchased from Thermo Fisher (Waltham, MA, USA).

### 3.2. Sample Preparation 

#### 3.2.1. Cell Lines Culture 

Human breast cancer epithelial cell lines MDA-MB-231 (HTB26) and MCF-7 (HTB-22) were purchased from ATCC, USA, and KAIMRC1 cells were established in our laboratory. All the cells were maintained in advanced Dulbecco’s Modified Eagle Medium (DMEM) supplemented with 10% Fetal Bovine Serum (FBS), 50 units/mL penicillin and 50 μg/mL streptomycin (Gibco), and 2 mM L-glutamine (Gibco). Cells were cultured at 37 °C in a humidified 5% CO_2_ atmosphere. 

#### 3.2.2. Confocal Laser Scanning Microscopy (cLSM)

cLSM of stained cells was performed using a Zeiss LSM 780 (Carl Zeiss, Jena, Germany) instrument equipped with argon and In-tune lasers. Cell Tracker™ Green (Life Technologies) was detected using Argon laser at 488 nm/520–530 (ex/em), and HOECHST 33342 (Thermo Fisher Scientific) was detected using UV laser at 350 nm/460 nm (ex/em).

#### 3.2.3. Protein Precipitation and Digestion

The frozen cellular pellets of the collected cell lines (KAIMRC1, MCF-7, and MDA-MB-231) were mechanically disrupted followed by resuspension in a mixture of RIPA buffer (Thermo-Scientific) and protease inhibitors (Sigma-Aldrich); 400 µg of proteins were precipitated for TMT and Phosphopeptide enrichment analysis using Methanol/chloroform/water. Proteins samples were then centrifuged, and pellets were collected. Proteins pellets were dissolved in 8 M urea/1 M triethylammonium bicarbonate. Subsequently, proteins were reduced and alkalied with 1 M Tris (2-carboxyethyl) phosphine hydrochloride and 500 mM 2-chloroacetamide, respectively. The digestion step was performed at 37 °C overnight using trypsin with a ratio of 1:40 (protein to trypsin). 

### 3.3. TMT Peptide Labeling 

Two biological replicates for each cell line in the absence or presence of FBS were labeled with TMT 6 plex following the manufacturer’s procedure. Briefly, 41 µL of anhydrous acetonitrile was added to each 0.8 mg TMT reagent and left for 5 min in room temperature to be completely dissolved. The digested peptides samples were then added to the correspondent TMT vial and then incubated for one hour at room temperature. Afterward, the reaction was quenched by adding 8 µL of 5% hydroxylamine. The labeled peptides in all TMT 6 channels were first equally combined and then split into 2 samples: (1) for high PH reverse-phase peptide fractionation prior to LC-MS/MS and (2) for TiO_2_ phosphopeptide enrichment analysis. 

### 3.4. High pH Reverse-Phase Peptide Fractionation 

Each TMT pooled labeled sample was fractionated offline on the basis of high pH (basic) reversed-phase chromatography prior to LC-MS/MS. Following the manufacture instruction, peptide sample was fractionated into 8 fractions using Pierce™ High pH Reversed-Phase Peptide Fractionation Kit (Thermo). Briefly, TMT samples were first evaporated using a speed vacuum and then resuspended with 0.1% formic acid. Reversed-phase fractionation spin columns were first conditioned with acetonitrile and 0.1% formic acid twice. The sample then loaded to the column and centrifuged at 3000× *g* for 2 min. Subsequently, the column was washed with water and then centrifuged at speed of 3000× *g* for 2 min. Finally, the sample was eluted into 8 fractions by changing the ratio of acetonitrile and 0.1% triethylamine. 

### 3.5. TiO_2_ Phosphopeptide Enrichment

The enrichment of phosphopeptide was performed on lyophilized TMT-labeled peptide samples following the manufacturer’s procedure in High-Select™ TiO_2_ Phosphopeptide Enrichment Kit. Briefly, the sample was first suspended in binding/equilibration buffer. TiO_2_ spin tip was washed and equilibrated with wash and binding/equilibration buffers followed by centrifugation for 2 min at speed of 3000× *g*. Labeled peptide sample was then loaded to TiO_2_ spin tip and spun at 1000× *g* for 2 min; the step was repeated twice. Subsequently, TiO_2_ spin tip was washed twice with binding/equilibration and wash buffer. Finally, phosphopeptide elution buffer was added to the spin tip and centrifuged for 5 min at 1000× *g*. The sample was dried and resuspended with 0.1% formic acid to be subjected to LC-MS/MS. 

### 3.6. NanoLC-MS/MS Analysis 

The TMT-labeled samples were analyzed on an Orbitrap Fusion Tribrid mass spectrometer (Thermo). Samples were injected directly onto a 25-cm 100 μm ID column packed with BEH 1.7 μm C18 resin (Waters). Samples were separated at a flow rate of 200 nL/min on an nLC 1000 (Thermo). Buffer A and B were 0.1% formic acid in water and acetonitrile, respectively. A gradient of 1–30% B over 160 min, an increase to 90% B over 60 min, and holding at 90% B for 20 min was used for a 240-min total run time. The column was re-equilibrated with 20 μL of buffer a prior to the injection of the sample. Peptides were eluted directly from the tip of the column and nanospray directly into the mass spectrometer by application of 2.8 kV voltage at the back of the column. The fusion was operated in a data-dependent mode. Full MS1 scans were collected in the Orbitrap at 120k resolution. The cycle time was set to 3 s, and within this 3 s, the most abundant ions per scan were selected for Collision-induced dissociation (CID) MS/MS in the ion trap. MS3 analysis with multi-notch isolation (SPS3) was utilized for the detection of TMT reporter ions at 30k resolution. Monoisotopic precursor selection was enabled, and dynamic exclusion was used with exclusion duration of 10 s.

### 3.7. Western Blot Analysis

MCF-7, MDA MB-231, and KAIMRC1 cells were seeded in 6-well plates in complete DMEM for 48 h. Prior to protein extraction, cells were preincubated with 10% serum-containing complete DMEM and serum-free DMEM for 24 h. The western blotting analysis was performed with rabbit polyclonal antibody against MTAP (Cat # PA5-87938, Invitrogen; 1:500), rabbit polyclonal antibody against KCTD12 (Cat # PA5-68689; 1:500), mouse monoclonal antibody to PARP (Clone 123; Cat # 436400, Thermo Fisher Scientific; 1:500), mouse monoclonal antibody to Lamin A/C (mab636; Cat # MA3-1000, Invitrogen; 1:500), rabbit polyclonal antibody to Phospho-Lamin A/C (Ser22; Cat # PA5-17113, Invitrogen; 1:1000), and rabbit polyclonal antibody to Phospho-Lamin A (Ser392; Cat # PA5-38290, Invitrogen; 1:1000). Signals were detected using a ChemiDoc MP System (Bio-Rad) and analyzed on ImageLab software. Sample loading was examined by probing with anti-β-actin antibody (Cell Signaling).

### 3.8. Protein Identification and Quantification and Data Processing

Peptide/protein identification and quantification were performed using Integrated Proteomics Pipeline–IP2 (Integrated Proteomics Applications). The raw mass spectrometry proteomics data have been deposited to the ProteomeXchange Consortium via the Proteomics Identification Database-EMBL-EBI (PRIDE) [49] partner repository with the dataset identifier PXD017721. The MS raw data files were converted into mzXML format using RawConverter [50]. For protein identification, tandem mass spectra were searched against a database including the Uniprot human database, reversed sequences, and contaminate using ProLuCID [51]. The search was set with 50 and 600 ppm for precursor and fragments mass tolerance, respectively. The precursor mass range was set from 600 to 6000 and selected trypsin as a protease enzyme. The N-term static modification was considered as (+229.1629) for TMT labeling, and the amino acid residue-specific static modifications were (+57.02146) on cysteine for carbamidomethylation and (+229.1629) on lysine for TMT labeling. The differential modification in the phosphopeptide enrichment analysis was set as (+79.9663) on serine, threonine, and tyrosine for phosphorylation. Identified proteins were further filtered with 1% false discovery rate (FDR) using DTASelect [52]. Protein quantitative analysis was achieved by Census tool [53]. The statistical analysis for the quantitative results was done by quantitative COMPARE tool, part of IP2. 

Go enrichment analysis for protein classification was performed using Protein Analysis THrough Evolutionary Relationships (PANTHER) system. The pathway enrichment analysis was generated using the Database for Annotation, Visualization, and Integrated Discovery (DAVID) software. For treemap and Volcano plots visualization, Tableau software was used (v. 2018.1.1, Tableau, WA, USA). Protein–protein interaction network analysis was generated using the Search Tool for the Retrieval of Interacting Genes/Proteins (STRING) database. 

## 4. Conclusions

In this study, we have performed a complete protein and phosphoprotein profiling of KAIMRC1 cell line in comparison to MDA-MB-231 and MCF-7 cell lines. Our results revealed that a number of proteins are differentially expressed, with a high degree of significance, among the three studied cell lines. Interestingly, the majority of the differentially expressed proteins are known to be involved in different cancer types including breast cancer. Pathway analysis of the differently expressed proteins in KAIMRC1 cells resulted in the identification of altered regulation of several breast cancer-associated pathways, including DNA replication, mismatch repair, apoptosis, and focal adhesion pathways. Interestingly, the majority of the identified pathways were associated with the upregulated phosphoproteins in KAIMRC1 during the starvation condition that includes antigen processing and presentation and the estrogen signaling pathway. One of the highlights of this proteomics-based investigation is the uncovering of the overexpression of MTAP, KCTD12, and PAPRP1 in the KAIMRC1 cells. We believe that our proteomics data in conjunction with ongoing genomics data will complement the characterization of this newly established cell line. These findings make KAIMRC1 cells a potential model to be used for screening these targets as anticancer drugs. In conclusion, our results strongly suggest that KAIMRC1 cells can be used as an alternative and unique model to study breast cancer-associated pathways in vitro.

## Figures and Tables

**Table 1:** Summary of the number of identified proteins and phosphoproteins in each cell line.

Cell Lines	Number of Proteins	Number of Phosphoproteins
KAIMRC1	5803	617
MCF-7	5993	674
MDA-MB-231	5747	545

## Supplementary Materials

Supplementary materials can be found at https://www.mdpi.com/1422-0067/21/12/4328/s1.
