# Supplementary material for: Proteomics Profiling of KAIMRC1 in Comparison to MDA-MB231 and MCF-7
Source: Int J Mol Sci. 2020 Jun 18;21(12):4328. doi: 10.3390/ijms21124328 (PMC7352455; doi:10.3390/ijms21124328)
Supplement: Supplementary file 1 [file ijms-21-04328-s001.zip › ijms-706332supplementry/Supporting Information-REVIESD 2.pdf]

Supplementary Material

Proteomics Profiling of KAIMRC1 in Comparison to MDA-MB231 and MCF-7

Bandar Alghanem [1], Rizwan Ali [1], Atef Nehdi [1], Hajar Al Zahrani [1], Abdulelah Altolayyan [1], Hayat Shaibah [1], Omar Baz [1], Alshaimaa Alhallaj [1], James J. Moresco [2], Jolene K. Diedrich [2], John R. Yates III [2] and Mohamed Boudjelal [1,*]

[1] Medical Research Core Facility and Platforms (MRCFP), King Abdullah International Medical Research Center/ King Saud bin Abdulaziz University for Health Sciences (KSAU-HS), King Abdulaziz Medical City (KAMC), NGHA, Riyadh 11426, Saudi Arabia

[2] Department of Molecular Medicine, The Scripps Research Institute, La Jolla, CA 92037, USA

* Correspondence: boudjelalmo@ngha.med.sa

Table S1 List of phosphopeptides with phospho-site localization for KAIMRC1 vs. MCF7 and MDA-MB- 231 in the presence of FBS

Table S2 List of phosphopeptides with phospho-site localization for KAIMRC1 vs. MCF7 and MDA-MB- 231 in the absence of FBS

Table S3 List of Differentially Expressed Proteins for KAIMRC1 vs. MCF7 and MDA-MB-231in the presence or absence of FBS. Statistical criteria with set with estimated fold change ≥ 2 for up-regulation and ≤ 0.5 for down-regulation with p value <0.05

Table S4 List of Differentially Expressed PhosphoProteins for KAIMRC1 vs. MCF7 and MDA-MB- 231 in the presence or absence of FBS. Statistical criteria with set with estimated fold change ≥ 2 for up-regulation and ≤ 0.5 for down-regulation with p value <0.05

Table S5 List of pathway terms for KAIMRC1 vs. MCF7 and MDA-MB-231in the presence or absence of FBS. P value < 0.05

Table S5 List of Differentially Expressed Proteins for KAIMRC1 vs. MCF7 and MDA-MB-231in the presence or absence of FBS. Statistical criteria with set with estimated fold change ≥ 5 for up-regulation and ≤ 0.2 for down-regulation with p value <0.001

Table S6 List of Differentially Expressed PhosphoProteins for KAIMRC1 vs. MCF7 and MDA-MB- 231 in the presence or absence of FBS. Statistical criteria with set with estimated fold change ≥ 5 for up-regulation and ≤ 0.2 for down-regulation with p value <0.01

Tables S1, S2, S3, S4, S5, and S6 can be found in supplemental Excel file called:

"Table_S1.xlsx"

"Table_S2.xlsx"

"Table_S3.xlsx"

"Table_S4.xlsx"

"Table_S5.xlsx"

"Table_S6.xlsx"

Results were generated from IP2 - Integrated Proteomics Pipeline Ver. 6.0.5.

A)

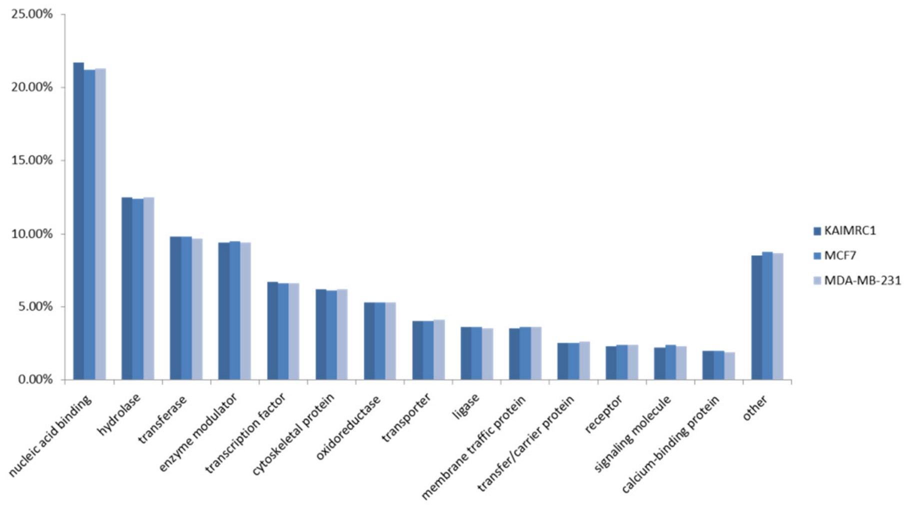

B)

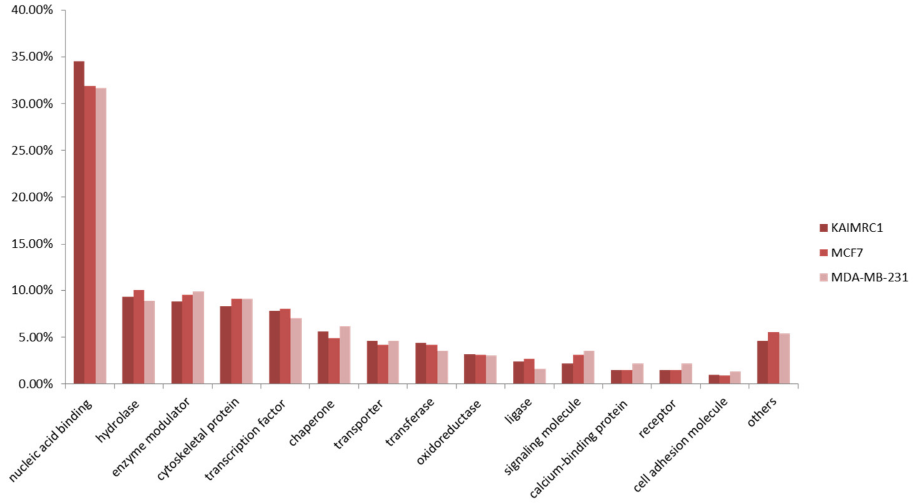

Figure S1. Classification of identified proteins based on protein class.

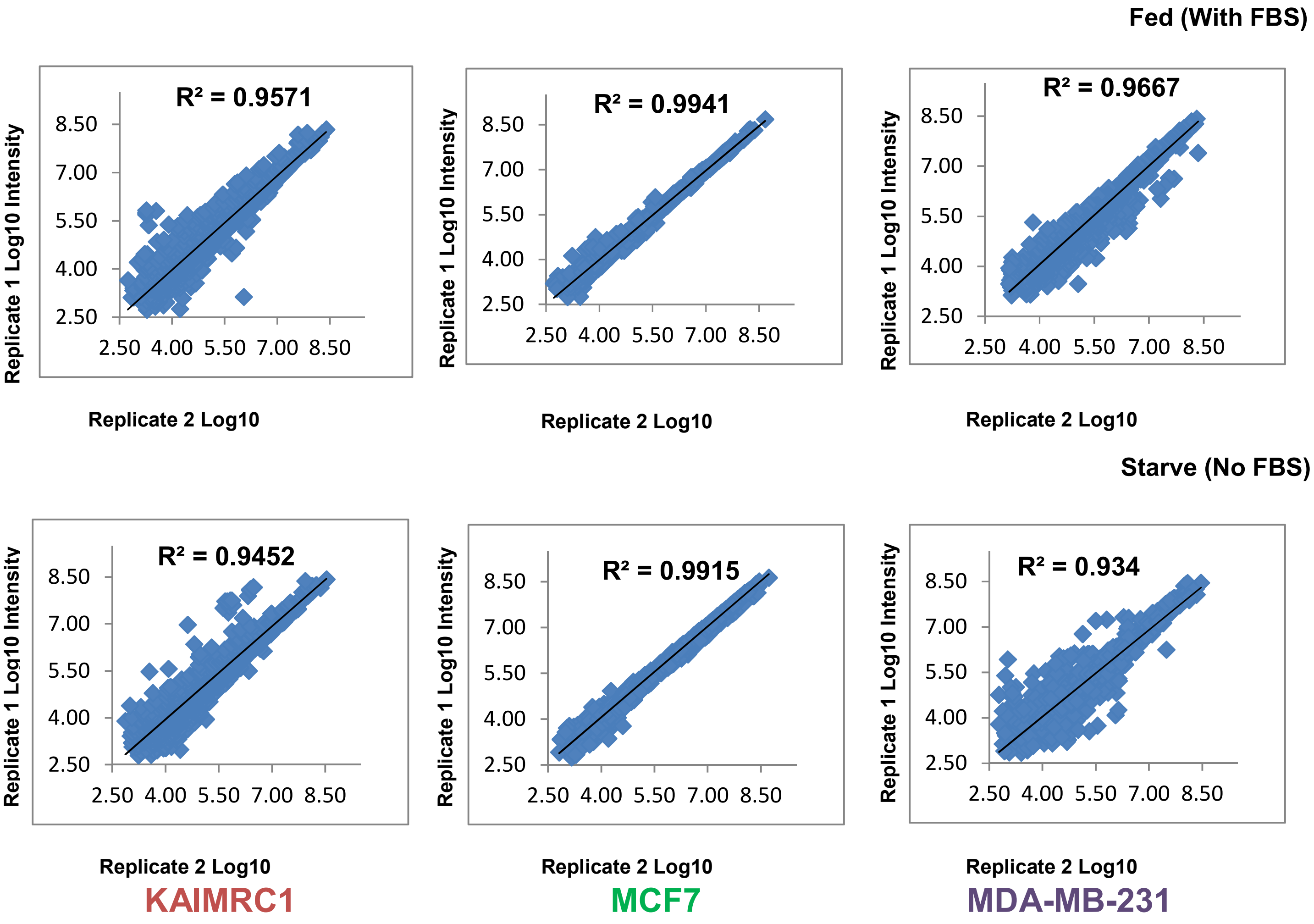

Figure S2. Reproducibility evaluation of proteins for duplicate biological replicates in KAIMRC1, MCF-7, and MDA-MB-231 cell lines using proteins normalized intensities, (top) cells grown in normal conditions, (bottom) under serum starvation.

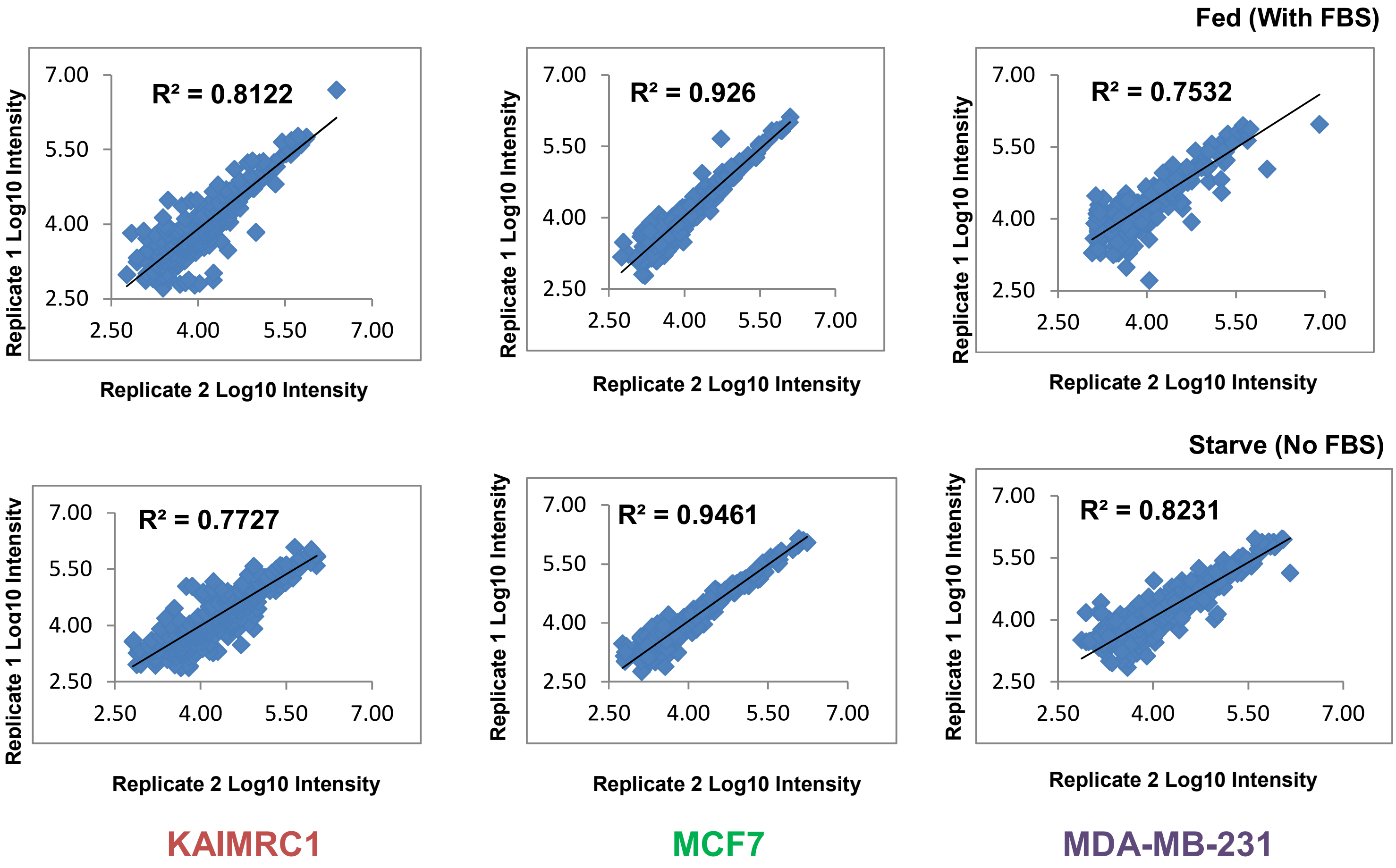

Figure S3. Reproducibility evaluation of Phospoproteins for duplicate biological replicates in KAIMRC1, MCF-7, and MDA-MB-231 cell lines using proteins normalized intensities, (top) cells grown in normal conditions , (bottom) under serum starvation.

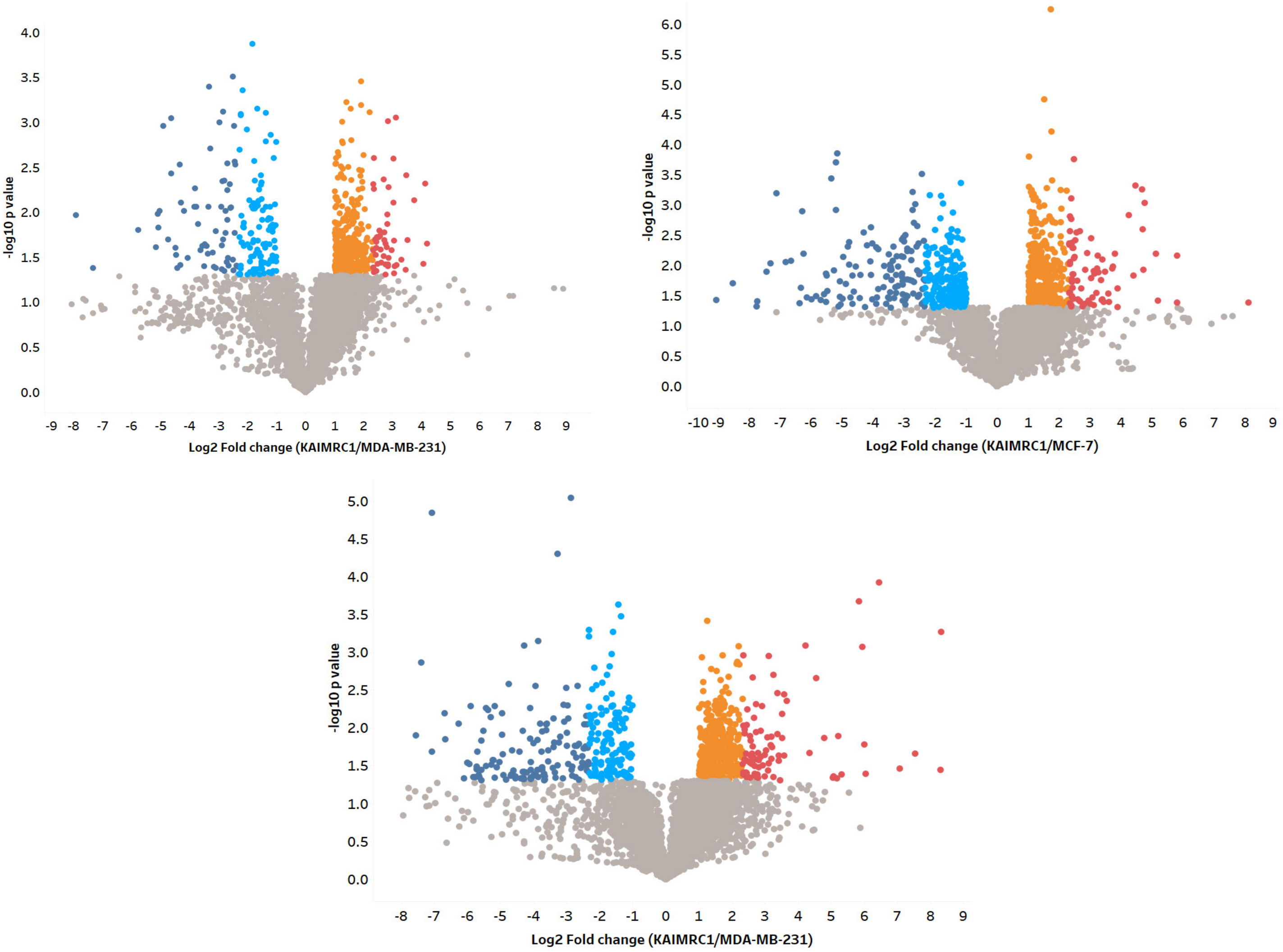

Figure S4. Volcano plot display differentially expressed proteins. Colored circles are significant proteins (P value < 0.05), Red is highly up regulated proteins with fold change ≥ 5. Up-regulated protein plotted in orange with fold change between 2 to 5. Light blue, down regulated protein with fold change ≤ 0.5. Dark blue, highly down regulated protein with fold change ≤ 0.2. Gray circles non-significant proteins. (Top) cells grown in normal conditions, (bottom) under serum starvation.

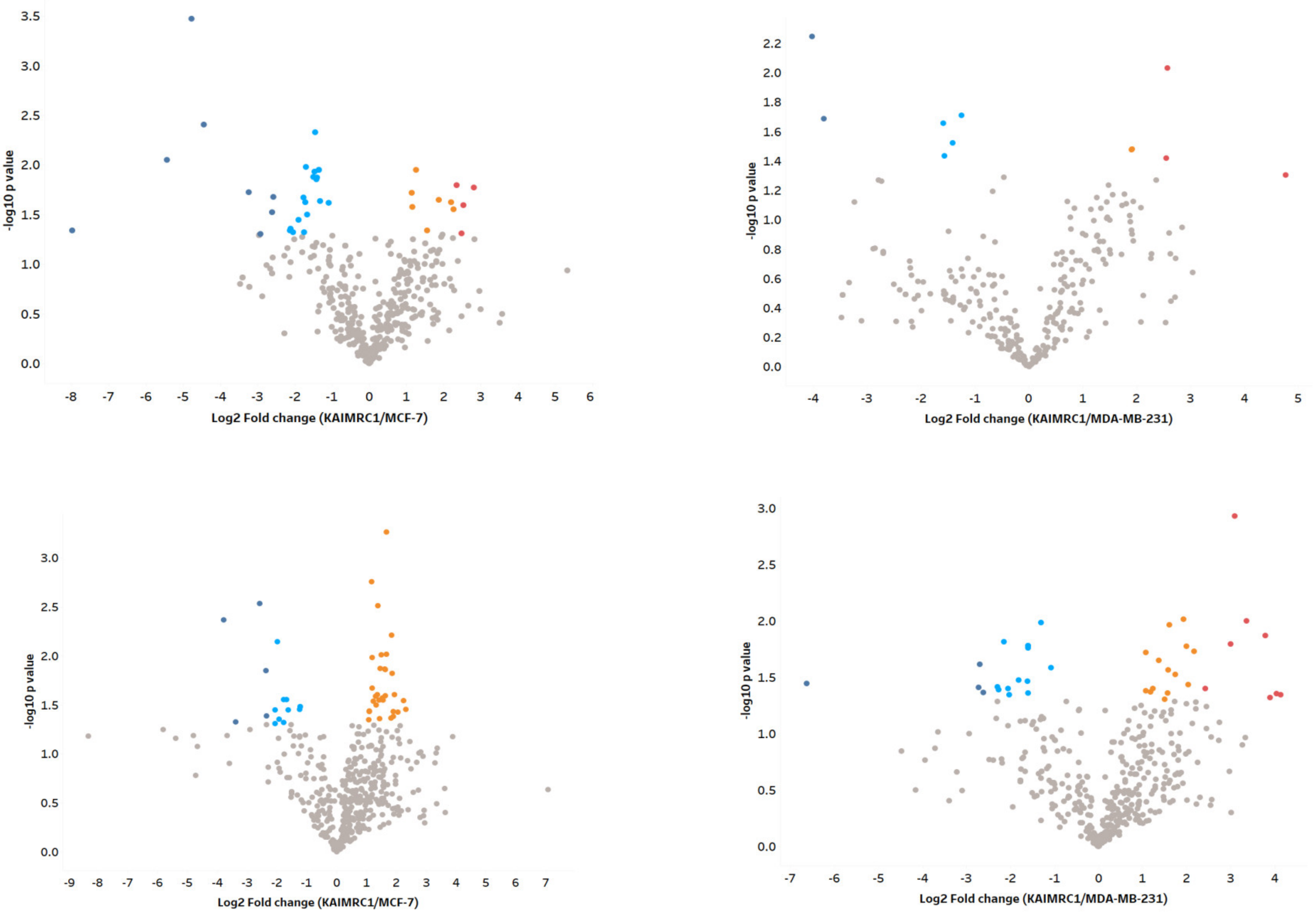

Figure S5. Volcano plot display differentially expressed Phosphoproteins. Colored circles are significant proteins (P value < 0.05), Red is highly up regulated proteins with fold change ≥ 5. Up-regulated protein plotted in orange with fold change between 2 to 5. Light blue, down regulated protein with fold change ≤0.5. Dark blue, highly down regulated protein with fold change ≤ 0.2. Gray circles non-significant proteins. (Top) cells grown in normal conditions, (bottom) under serum starvation.
