# Supplementary figures and images for: Proteomics Profiling of KAIMRC1 in Comparison to MDA-MB231 and MCF-7
Source: Int J Mol Sci. 2020 Jun 18;21(12):4328. doi: 10.3390/ijms21124328 (PMC7352455; doi:10.3390/ijms21124328)

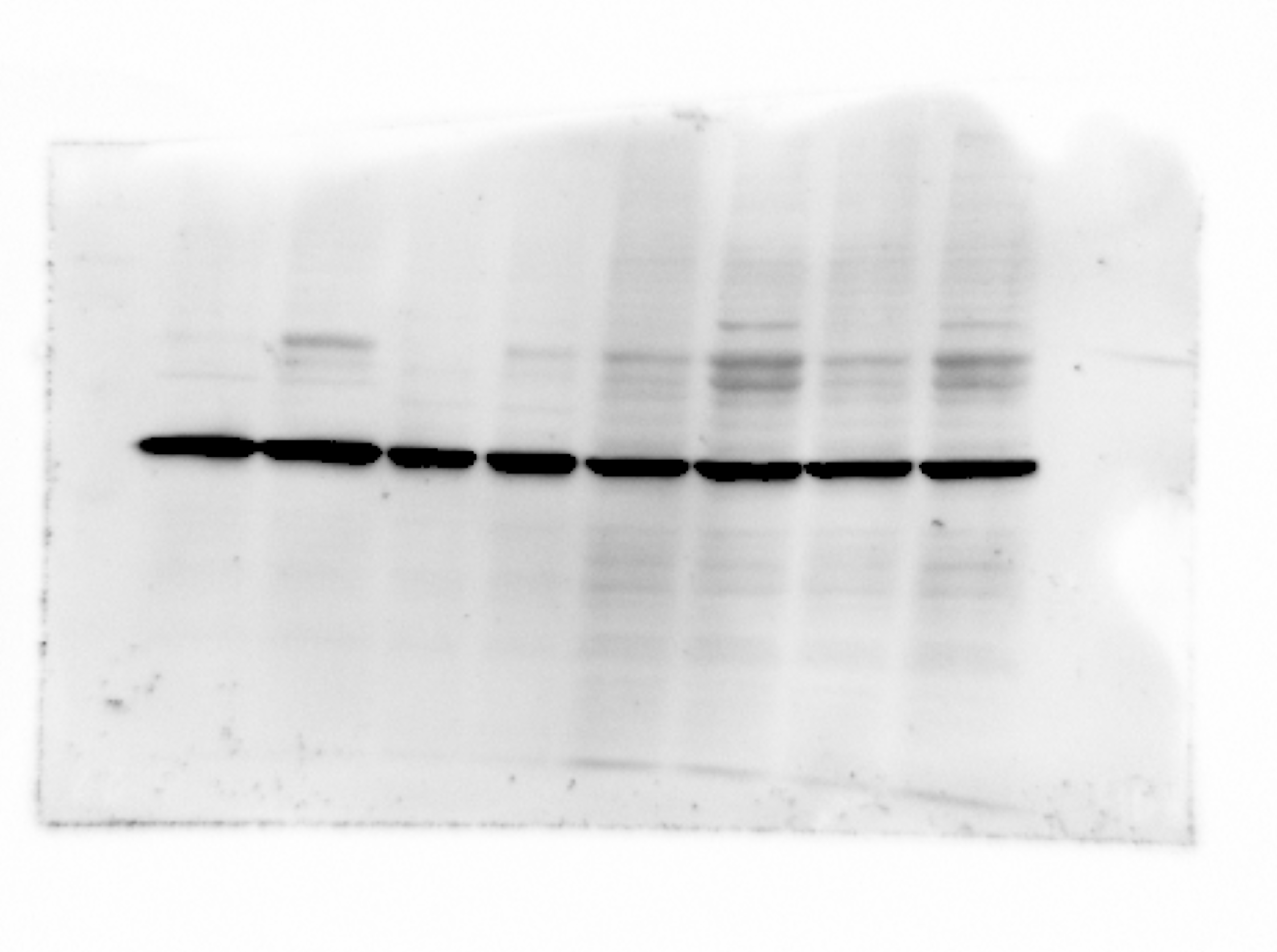

Supplement: Supplementary file 1 [file ijms-21-04328-s001.zip › ijms-706332supplementry/western blot-original images/bactin WB.tif]

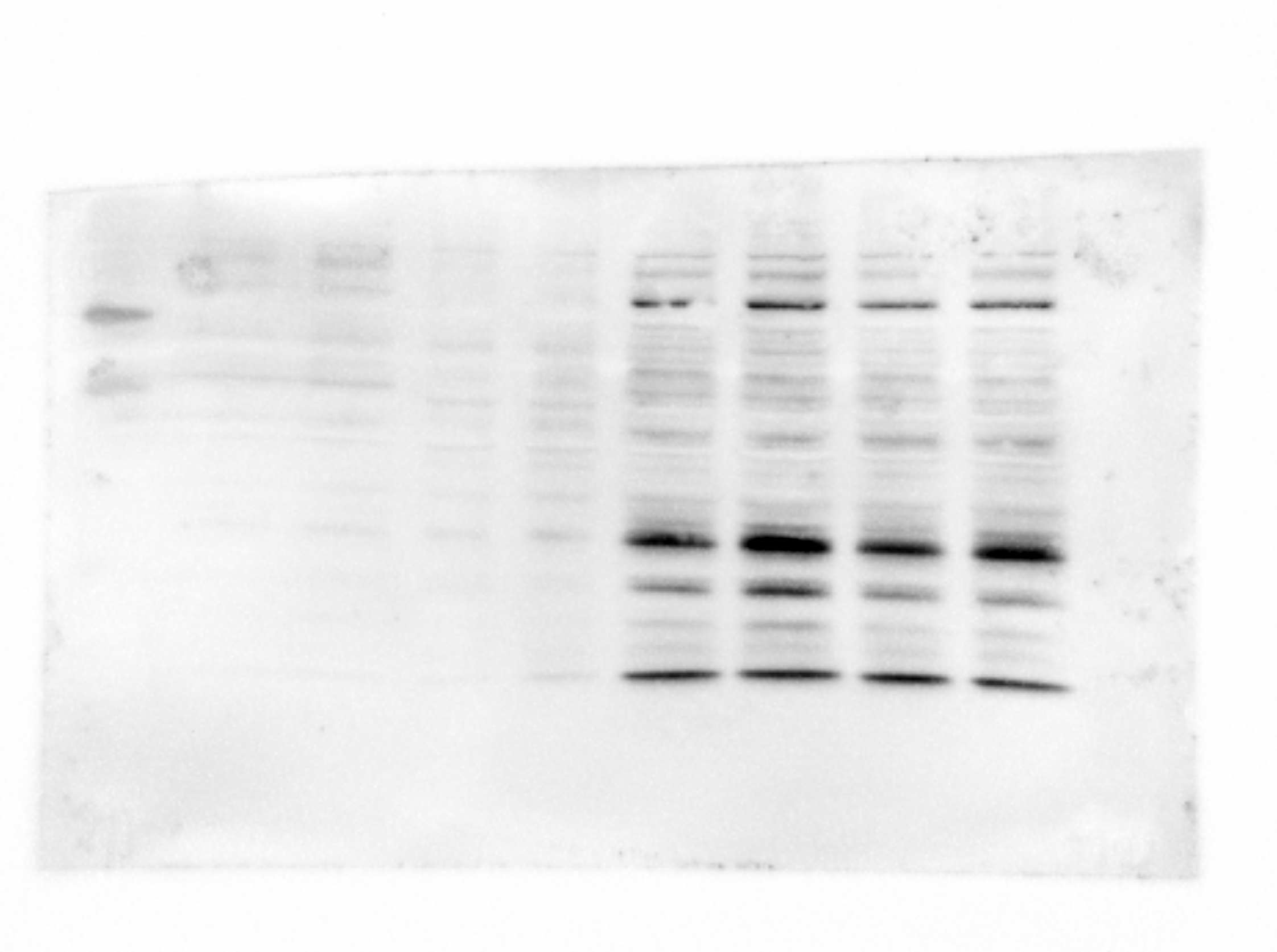

Supplement: Supplementary file 1 [file ijms-21-04328-s001.zip › ijms-706332supplementry/western blot-original images/KCTD WB.tif]

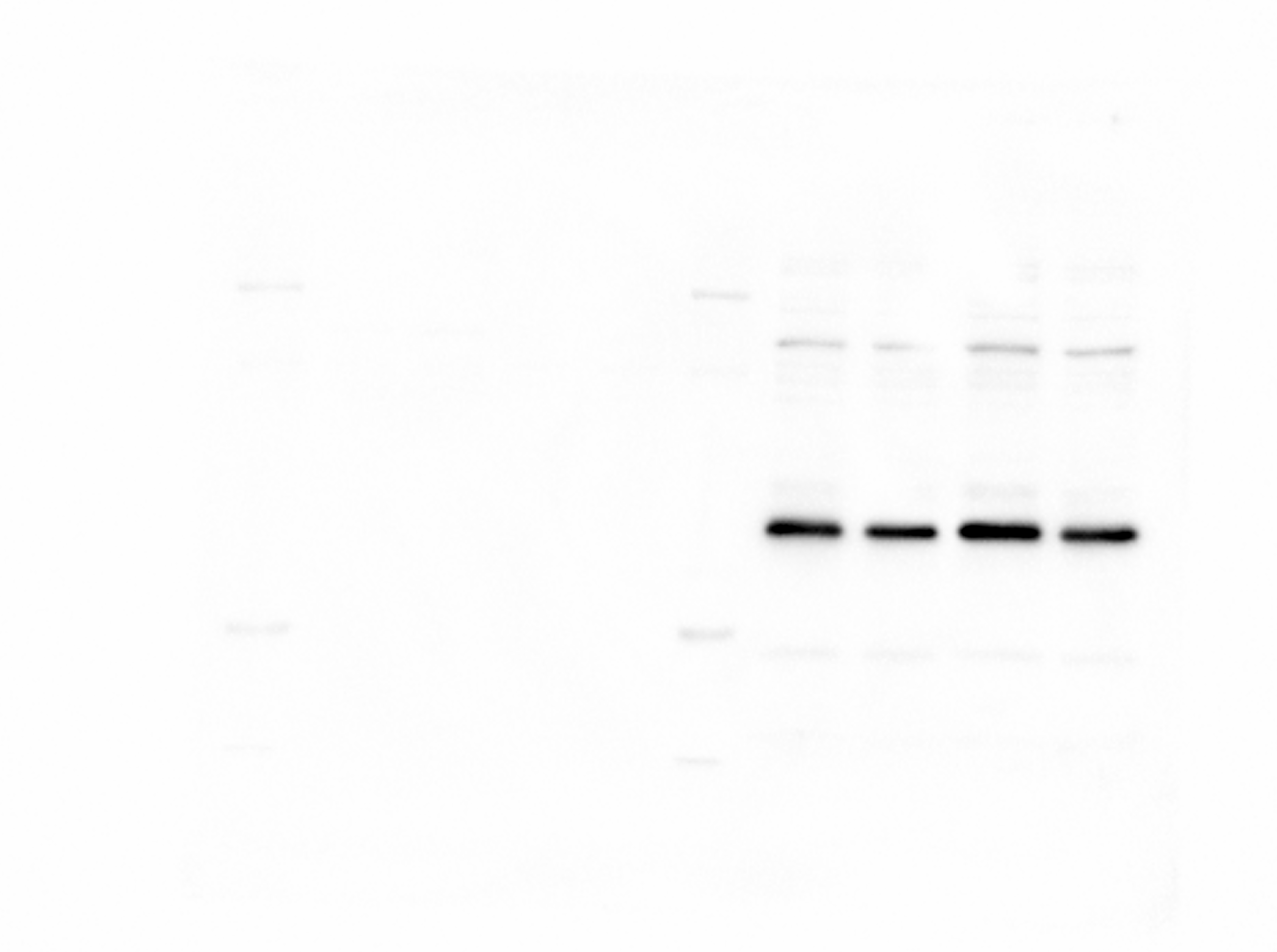

Supplement: Supplementary file 1 [file ijms-21-04328-s001.zip › ijms-706332supplementry/western blot-original images/MTAP WB.tif]

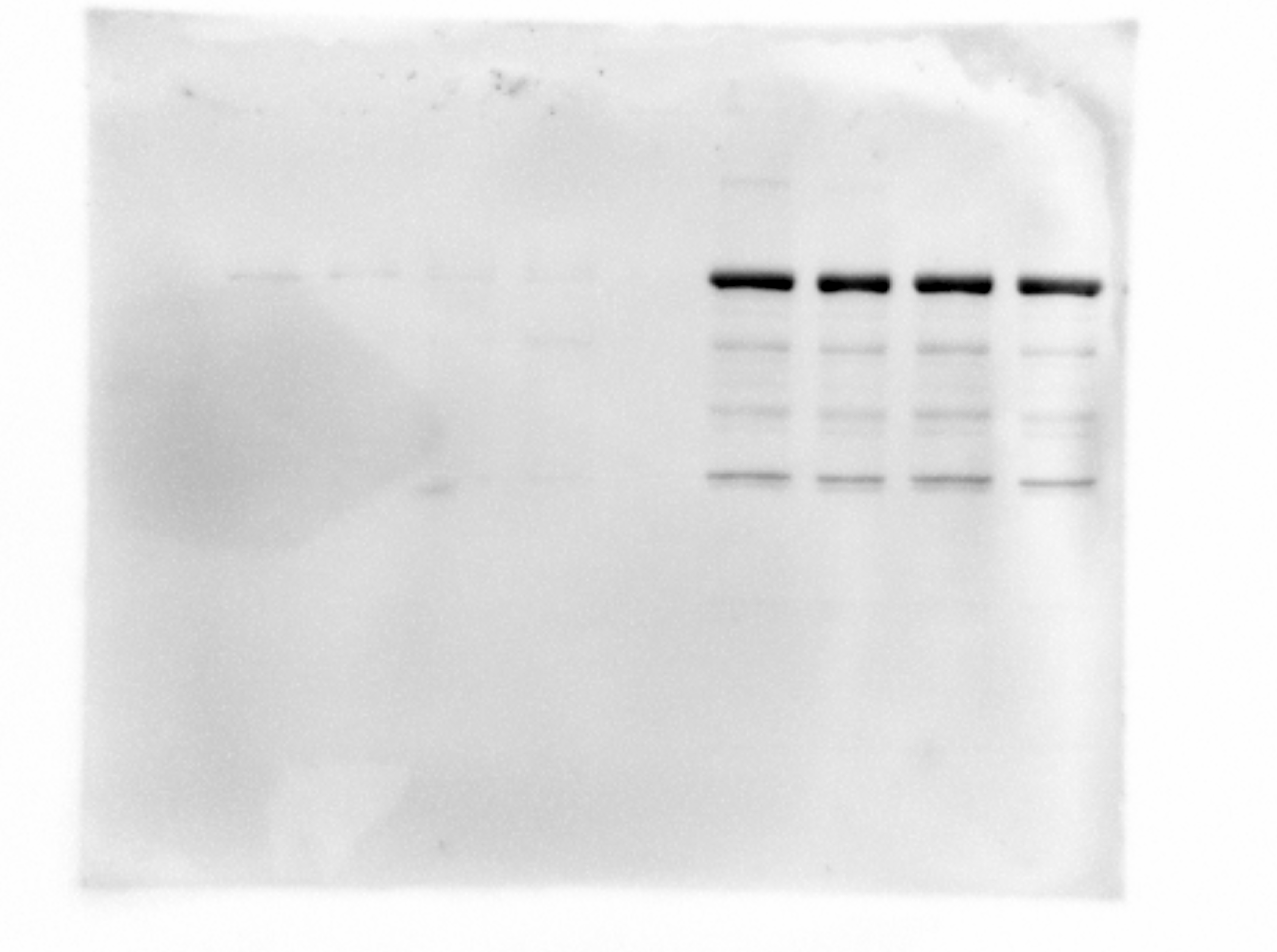

Supplement: Supplementary file 1 [file ijms-21-04328-s001.zip › ijms-706332supplementry/western blot-original images/PARP WB.tif]

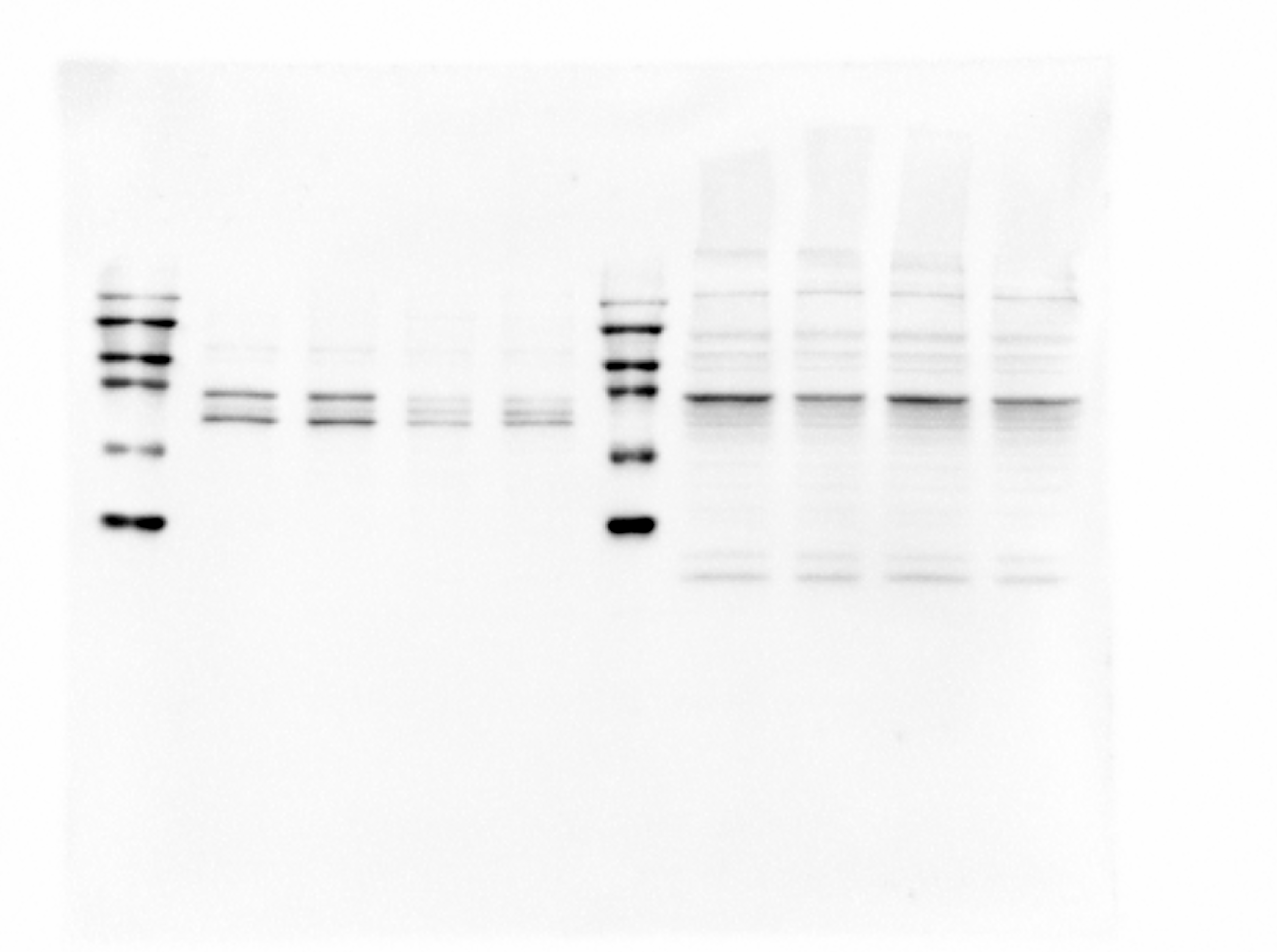

Supplement: Supplementary file 1 [file ijms-21-04328-s001.zip › ijms-706332supplementry/western blot-original images/pLamin SER 392 WB.tif]

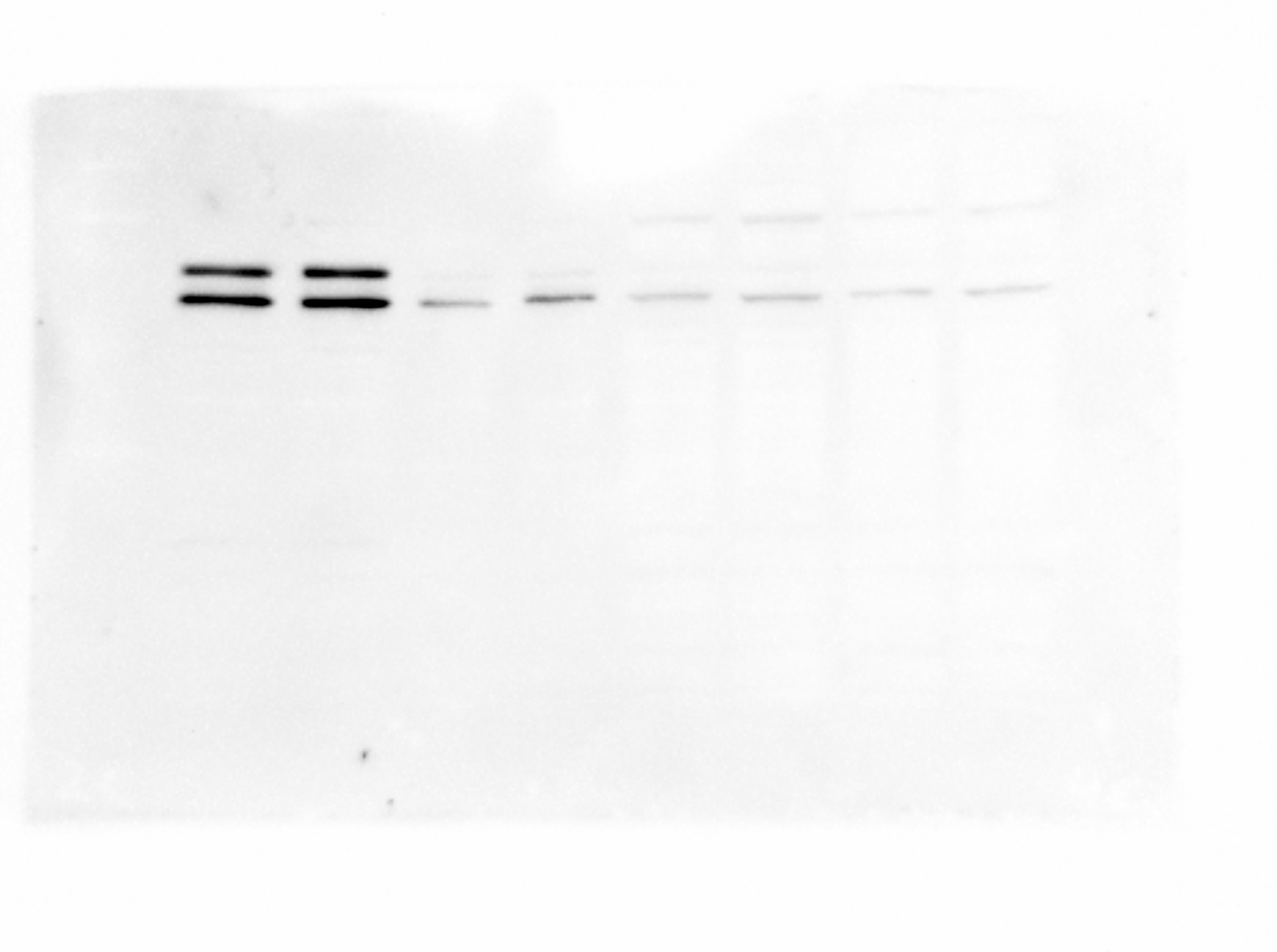

Supplement: Supplementary file 1 [file ijms-21-04328-s001.zip › ijms-706332supplementry/western blot-original images/pLamin WB.tif]
